# Early Monitoring of Response (MORE) to Golimumab Therapy Based on Fecal Calprotectin and Trough Serum Levels in Patients With Ulcerative Colitis: A Multicenter Prospective Study

**DOI:** 10.2196/resprot.5791

**Published:** 2016-06-28

**Authors:** Attyla Drabik, Andreas Sturm, Margit Blömacher, Ulf Helwig

**Affiliations:** ^1^ Clinical Trial Support Münster Germany; ^2^ DRK Kliniken Berlin Westend Department of Gastroenterology Berlin Germany; ^3^ Kompetenznetz Darmerkrankungen e. V. Kiel Germany; ^4^ Medical Practice for Internal Medicine Oldenburg Oldenburg Germany

**Keywords:** ulcerative colitis, golimumab, fecal calprotectin, tumor necrosis factor alpha

## Abstract

**Background:**

The treatment of ulcerative colitis (UC) patients with moderate to severe inflammatory activity with anti-tumor necrosis factor alpha (TNFα) antibodies leads to a clinical remission rate of 10% after 8 weeks of therapy. However, it must be taken into account that patient selection in clinical trials clearly influences both response and remission rates. An unsatisfactory response to anti-TNFα medication after week 12 often leads to a discontinuation of treatment. The early prediction of clinical response could therefore help optimize therapy and potentially avoid ineffective treatments.

**Objective:**

The aim of this study is to develop an algorithm for optimizing golimumab administration in patients with moderate to severe UC by calculating the probability of clinical response in Week 26 based on data from Week 6.

**Methods:**

The study is designed as a prospective, single-arm, multicenter, non-interventional observational study with no interim analyses and a sample size of 58 evaluable patients. The primary outcome is the prediction of clinical response in Week 26 based on a 50% reduction in fecal calprotectin and a positive golimumab trough level in Week 6.

**Results:**

Enrollment started in October 2014 and was still open at the date of submission. The study is expected to finish in December 2016.

**Conclusions:**

The early identification of patients who are responding to an anti-TNFα antibody is therapeutically beneficial. At the same time, patients who are not responding can be identified earlier. The development of a therapeutic algorithm for identifying patients as responders or non-responders can thus help prescribing physicians to both avoid ineffective treatments and adjust dosages when necessary. This in turn promotes a higher degree of treatment tolerance and patient safety in the case of anti-TNFα antibody administration.

**ClinicalTrial:**

German Clinical Trials Register, Deutsches Register Klinischer Studien DRKS00005940; https://drks-neu.uniklinik-freiburg.de/drks_web/navigate.do?navigationId=trial.HTML&TRIAL_ID=DRKS00005940 (Archived by WebCite at http://www.webcitation.org/6i4Xoo1sH)

## Introduction

### Background

The treatment of ulcerative colitis (UC) patients with moderate to severe inflammatory activity with anti-tumor necrosis factor alpha (TNFα) antibodies leads to a clinical remission rate of 10% (ULTRA1 study, 80/40 mg adalimumab) [1] to 39% (ACT1 study, infliximab 5 mg/kg body weight [2] and SUCCESS study [3]) after 8 weeks of therapy. However, it must be taken into account that patient selection in clinical trials clearly influences both response and remission rates. In addition, the results of these studies are not consistent with those of observational trials with a high degree of target population representativeness. International guidelines therefore recommend that the success of an anti-TNFα therapy beyond the 12-week mark must be predicted as soon as possible in order to adjust or discontinue treatment depending on the clinical situation [1,4-6]. An unsatisfactory response to anti-TNFα medication after Week 12 often leads to a discontinuation of treatment. The early prediction of clinical response could therefore help to optimize therapy and potentially avoid ineffective treatments [7].

In this regard, the PURSUIT study showed that the administration of the new human anti-TNFα immunoglobulin G1 monoclonal antibody golimumab to subjects with moderately to severely active UC induced clinical response in Week 6 after two injections (Weeks 0 and 2) in 51.0-54.9% of patients in a randomized clinical trial, whereas placebo response was 30.3% (*P*<.001) [8].

After 6 weeks, responders in the golimumab group were randomized again to receive either placebo or 50 or 100 mg golimumab doses every 4 weeks for a further 54 weeks. The analysis of clinical response in this trial showed a statistically significant response for golimumab 50 mg versus placebo (*P*<.001) after 54 weeks (47.0% and 31.2% respectively) [9].

### Study Objective

This early Monitoring of Response (MORE) study (DRKS00005940) seeks to achieve a more thorough understanding of therapeutic development in patients with moderate to severe ulcerative colitis receiving regular doses of golimumab. The aim is to develop an algorithm for optimizing golimumab administration in patients with moderate to severe ulcerative colitis by calculating the probability of clinical response in Week 26 based on data from Week 6.

## Methods

### Trial Design

The study is carried out in conformity with the German Medicinal Products Act (Arzneimittelgesetz, AMG) and is a non-interventional study in accordance with the Medicinal Products Act (§ 4 section 23 p. 3 AMG). The study is designed as a prospective, single-arm, multicenter, non-interventional observational study with no interim analyses and a sample size of 58 evaluable patients, for which 61 patients must be recruited.

### Outcomes

#### Primary

The primary outcome is the prediction of clinical response in Week 26 based on a 50% reduction in fecal calprotectin and a positive golimumab trough level of >2.5 μg/ml in Week 6.

Our definition of a clinical response is a reduction in the partial Mayo score by 2 points between baseline and Week 6, or a partial Mayo score of ≤1 in Week 6.

#### Secondary

In addition to the primary study goal, the following secondary outcomes are analyzed:

1. At which point in time is a 50% reduction in fecal calprotectin a reliable predictor of response?

2. How strong is the correlation between antibody towards golimumab (ATG) level and fecal calprotectin level?

3. How strong is the correlation between ATG and the partial Mayo score?

4. How strong is the correlation between golimumab trough level and fecal calprotectin?

5. How strong is the correlation between golimumab trough level and the partial Mayo score?

6. Do the following parameters exert any influence on clinical response?

      a) C-reactive protein (CRP)

      b) white blood cells (WBC) count

      c) hemoglobin

      d) platelet count

      e) ferritin

7. Frequency of adverse reactions

### Statistics

#### Analysis

Our aim is to study the detection of a positive trough level of golimumab of >2.5 μg/ml and a significant reduction in fecal calprotectin of 50% from baseline in Week 6 as a reliable predictor for clinical response in Week 26 during long-term treatment with golimumab. In this connection, a level between 2.5 μg/ml and 4.3 μg/ml is regarded as being associated with a therapeutic effect of golimumab [8,9].

Statistical analysis is mainly carried out with descriptive methods such as frequency tables, and statistical parameters such as mean, standard deviation, and quantiles. Bar graphs are used for qualitative data and box-and-whisker plots for quantitative data. In addition, inferential statistical analysis is carried out with relevant significance tests and confidence intervals. Missing data are not imputed.

In order to evaluate the primary outcome, the test problem presented in the section on statistical hypotheses is formulated and solved. The test is carried out with a power of 80% and a two-tailed significance level of alpha=.05. Analysis is carried out with a logistic regression model based on the percentage change of fecal calprotectin and golimumab trough levels. In addition, secondary parameters (eg, ATGs, CRP, WBC) are also analyzed to identify further correlations between the data of Week 6 and therapy outcome in Week 26. The aim is to identify further constraints to the statistical model in order to take into account all relevant parameters in the final version of the algorithm. Secondary outcomes are assessed in an explorative way, that is, no pre-formulated hypotheses are tested. The *P* values obtained are thus interpreted according to Fisher’s method: a *P* value is considered a metric value, and the smaller the *P* value, the larger the significance of the corresponding effect. No interim analyses are planned. Data analysis is carried out only once, at the end of the study.

#### Hypotheses

The following two-tailed test problem is used for the primary outcome:

Hypothesis 0: beta1=0 versus Hypothesis 1: beta1≠0, where beta1 is the coefficient of the logistic regression model, and null hypothesis: H0. There is no correlation between a significant reduction in fecal calprotectin of 50% from baseline in Week 6 and clinical response to therapy with golimumab in Week 26.

Therefore, our research hypothesis, H1, is that there is a correlation between a significant reduction in fecal calprotectin of 50% from baseline in Week 6 and clinical response to therapy with golimumab in Week 26.

### Sample Size

#### Rationale

Sample size is planned based on data from studies researching a correlation between fecal calprotectin and response to an anti-TNFα therapy.

De Vos et al [10] describe response rates as: “After 10 weeks anti-TNFα therapy induced endoscopic remission in 63% (confidence interval: 47–78%) of patients”. Molander et al [11] describe the correlation between the predictive quality of fecal calprotectin and the remission rate. The results are displayed in Table 1.

**Table 1:** Cross classification of fecal calprotectin predictive quality and remission.

		Fecal calprotectin decline		Total
		Yes	No	
Remission				
	Yes	30	3	33
	No	6	21	27
Total		36	24	60

Based on these results, the odds ratio (OR) is calculated as OR (30*21) / (6*3)=35. It should be noted that for the above study the cut-off point for fecal calprotectin decline was a reduction of >75% from baseline. Lower cut-off points, for example, a 50% reduction, would lead to smaller OR, as the number of patients with neither decline nor response is described as being almost constant in the above-mentioned literature: “Absence of decrease in calprotectin levels at week 6 identified patients resistant to the treatment” [11]. It is therefore assumed for sample size calculation that 80% of the patients will have a “fecal calprotectin decline.” Table 2 summarizes the scenarios that have been taken into consideration.

**Table 2:** Sample size rationale: Response rates and their effect on resulting OR for 9 different scenarios.

Scenario	Response rate (%)	OR
1	40	10
2	40	20
3	40	30
4	50	10
5	50	20
6	50	30
7	60	10
8	60	20
9	60	30

#### Calculation

Sample size calculation is carried out with the statistical analysis software SAS. Table 3 shows the required number of evaluable patients for each scenario. It is expected that 5% of the intention-to-treat principle population will be excluded. Sample size is inversely proportional to the OR and the response rate.

**Table 3:** Sample size calculation: Number of evaluable subject and total number of subjects considering dropouts for 9 different scenarios.

Scenario	Response rate, %	OR	Evaluable subjects, n	Subjects including potential dropouts, total n
1	40	10	58	61
2	40	20	40	42
3	40	30	34	36
4	50	10	50	53
5	50	20	34	36
6	50	30	29	31
7	60	10	45	48
8	60	20	31	33
9	60	30	26	28

To prevent study failure due to an underpowered study, a worst case scenario with a response rate of 40% and an OR of 10 is used as a basis for sample size. A total of 58 evaluable subjects are therefore necessary for the trial, thus 61 patients must be recruited.

### Study Population

The evaluation of primary and secondary outcomes is carried out according to the intention-to-treat principle. The corresponding population comprises all patients included in the study regardless of possible protocol violations (eg, dropouts). In addition to intention-to-treat analysis, sensitivity analysis according to the per-protocol principle is carried out. Relevant protocol violations leading to exclusion from the per-protocol group are defined in the statistical analysis plan.

### Selection of Study Centers

All study centers are part of the German Inflammatory Bowel Disease (IBD) Study Group and are chosen according to their main area of focus and their experience in the treatment of UC. By signing the investigator agreement, each study center selected confirms its fulfilment of all formal requirements for inclusion in the study and guarantees its compliance with data privacy laws and any other regulations pertaining to the execution of this observational study.

### Participant Criteria

The inclusion criteria for the study include (1) clinically and endoscopically confirmed diagnosis of UC, (2) study-independent treatment with golimumab according to current medical practices, (3) age ≥18 years, (4) sufficient German language communication skills, (5) ability of the patient to understand the nature, significance, and scope of the clinical trial and make an independent decision based on this knowledge, and (6) elevated calprotectin levels (≥100 mg/L or ≥100 mg/kg) within 3 weeks prior to inclusion.

Our exclusion criteria include (1) infectious colitis, (2) off-label treatment with golimumab, or (3) treatment with golimumab within the previous 3 months.

### Study-Specific Interventions

No medical interventions are carried out in the course of the study other than those required by standard medical procedure. When taking routine blood samples, golimumab serum levels and ATG levels should also be monitored if possible. Only the natural progress of the disease in UC patients is monitored and evaluated.

### Schedule of Visits

There are no defined study visits. In the course of the study, the only clinical and laboratory data recorded are those corresponding to standard medical procedure. Data are recorded in the following observational weeks: baseline, 2, 6, 14, 22, and 26. Deviations of +/- 3 days from this documentation schedule fall within the scope of the study protocol. The period until the next examination is subsequently shortened or lengthened accordingly in order to compensate for deviations and maintain the examination rhythm.

The following data are recorded at the initial screening/baseline examination: date of consent, screening date, inclusion and exclusion criteria, personal information (date of birth, sex, height, weight), date of initial diagnosis, information regarding prior anti-TNFα medication, partial Mayo score, laboratory tests (hemoglobin, WBC, platelet, ferritin, CRP, fecal calprotectin, and ATG), and medication (dose of golimumab, cortisone, and azathioprine).

The following data are recorded during each subsequent examination (week 2, 6, 14, 22, and 26) in the course of the study: partial Mayo score, laboratory tests (hemoglobin, WBC, platelet, ferritin, CRP, and fecal calprotectin), and medication (dose of golimumab, cortisone, and azathioprine). In addition, in Weeks 2 and 6, golimumab trough serum levels and ATG are measured.

### Documentation

Data are recorded using case report forms (CRF). The investigator is responsible for the timely, correct, complete, and legible recording of study data in the CRF and confirms recording by signature. CRF are completed with a black ballpoint pen. Corrections are documented as follows. The wrong entry is crossed out with a single line, and corrections are entered next to the crossed-out text and verified by date and initials, stating the reason for the change if necessary. Instructions for use (entry and corrections) are included in each CRF. Source data according to the ICH E6 guideline on good clinical practice (GCP) are original documents in patient files, as well as doctors’ letters, certified copies of original records, and laboratory printouts. In addition, all patient questionnaires (self-reporting) are also considered source data. Study data are to be recorded from patient files.

### Patient Identification

All patient data are pseudonymized. Each patient will be clearly identified by a patient identification number assigned at each study center. The investigator will keep a patient identification list documenting the patient identification number with the patient’s full name, date of birth, sex, and date of informed consent.

The patient identification list is part of the investigator file and will remain at the clinic. The patient identification number consists of a 2-digit clinic number, a hyphen, and a running 3-digit number of recruited patients per study center.

### Trial

#### Start of Patient Participation

Any patient with a clinically and endoscopically confirmed diagnosis of UC and qualified for golimumab treatment according to routine medical practice is a potential study candidate. All potential candidates who come to the attention of the investigator will be informed regarding the possibility of participating in the study. Potential candidates interested in participating in the study will be promptly informed about the study in order to obtain their informed consent in accordance with the section “Patient Information and Informed Consent”.

#### End of Patient Participation

The observation of each patient ends according to the schedule with the last study visit. A patient’s participation in the study will be terminated prematurely if at least one of the following criteria is met: (1) withdrawal of informed consent, (2) termination of golimumab treatment, (3) lack of medical justification for further participation in the study, (4) premature termination of the complete trial, or (5) subsequent discovery that not all inclusion criteria are met and/or that any exclusion criteria are met.

#### Trial Duration/End of Trial

The recruiting phase has a planned duration of 18 months. The observational phase has a planned duration of 26 weeks. The complete trial is considered to have ended after all queries from the study coordination center have been answered by each individual study center, but at the latest 4 months after the last visit of the last patient.

Study centers that grossly violate the AMG, data protection regulations, or the GCP guidelines can be excluded from the further recruitment and observation of study patients. Premature termination of the study as a whole will be taken into consideration if ethical or scientific justification for the trial is compromised or no longer valid, errors or violations significantly compromise the scientific integrity of the data collected for the study with regard to the study aims, or the requirements for a successful execution of the study are no longer fulfilled for other reasons.

The principal investigator will consult the corresponding biometrician regarding any possible premature termination of the trial. The minutes of the aforementioned consultation meeting will be recorded and subjected to the approval of both parties. Any decision regarding the premature termination of the trial will be taken jointly by the principal investigator and the corresponding biometrician.

### Data Quality Assurance

Upon receipt at the study coordination center, CRF will be checked for completeness and consistency (in-house review). Queries will be generated for missing or implausible entries and sent to the corresponding study centers. After the clarification of implausible entries and completion of missing data, CRF will be handed over to the corresponding data management department for data entry.

### Quality Control and Assurance

Quality control in the study is ensured by the possibility of monitoring the study centers involved. For each monitoring visit, a monitoring report is generated documenting the progress of the observational study and describing any problems that may have arisen. The exact nature and extent of the monitoring activities are described in a separate monitoring manual. All investigators declare their consent to regular visits of study monitors to the study centers. In addition, they must provide direct access to all necessary study documents, including original patient documents relevant to the study. The principal investigator and/or auditors designated by the principal investigator are entitled to conduct audits at the study centers and any other facilities participating in the study. They are entitled to inspect and review all study-relevant documents. This right also applies to regulatory inspectors.

### Ethical and Regulatory Aspects

The study is conducted in compliance with the current version of the Declaration of Helsinki (10/2013, Fortaleza, Brazil). This study cannot begin before approval has been obtained from the corresponding ethics committee. Prior to inclusion in the study, the investigator will inform each patient about the nature, significance, risks, and scope of the study, as well as the patient’s right to withdraw from the study at any time without prejudice. An informed consent form is handed to the patient describing the study in non-scientific and generally understandable language. Each patient must consent to study participation in writing. The patient must be provided with ample time to make a decision and given the opportunity to ask any questions before the consent form is signed. In accordance with AMG, § 40 Abs. 2a, patients are informed that the data related to their disease will be stored with a pseudonym and analyzed for scientific purposes. Patients must consent to the use of their pseudonymized data in writing. Informed consent forms are to be signed and dated by the patient and the treating physician.

This clinical study is carried out in conformity with the requirements of the current German Medicinal Products Act, as well as all applicable legal provisions regarding data protection and the GCP guidelines. The general notification requirement as per § 67 AMG will be complied with.

## Results

Enrollment started in October 2014 and was still open at the date of submission. The study is expected to finish in December 2016.

## Discussion

### Rationale for the Trial

The early identification of patients who are responding well to an anti-TNFα-antibody is therapeutically beneficial. At the same time, patients who are not responding can be identified earlier. The development of a therapeutic algorithm for identifying patients as responders or non-responders can help prescribing physicians both to avoid ineffective treatments and to adjust dosages when necessary. This in turn would promote a higher degree of treatment tolerance and patient safety in the case of anti-TNFα antibody administration.

There is a clear association between detectable trough levels of anti-TNFα inhibitors and clinical therapeutic response. On the other hand, the detection of anti-drug antibodies is associated with a poor response.

Fecal calprotectin levels reflect mucosal inflammation status. The strong correlation between high fecal calprotectin and mucosal inflammation has been widely described. Furthermore, it is also clear that fecal calprotectin levels decrease if there is therapeutic response.

A regular question among IBD patients is how long they will have to take their medication in total. Yet there is currently insufficient data regarding optimal treatment length with TNFα inhibitors. In view of the elevated costs and possible side effects associated with this type of treatment, it is desirable to clearly define a patient population for which continued therapy is justifiable.

### Justifications for Trial Design

This study is prospective, as the aim is to use the data from trial Week 6 to calculate the probability of clinical response in Week 26. Only one study cohort is required to answer this question, and no comparison group is necessary.

In order to achieve a higher degree of representativeness of the results for the intended population, the trial is carried out throughout Germany and at multiple centers specifically chosen for this purpose. Treatment, including diagnosis and monitoring, does not follow a predefined study protocol and is carried out exclusively in accordance with current medical practices. The aim of this non-interventional approach is to ensure a high degree of representativeness of the study results for daily medical practice.

### Conclusion

This early Monitoring of Response (MORE) study aims to achieve a more thorough understanding of therapeutic development in patients with moderate to severe ulcerative colitis receiving regular doses of golimumab by developing an algorithm for optimizing golimumab administration in patients with moderate to severe ulcerative colitis.

## Abbreviations

AMG: Arzneimittelgesetz, German Medicinal Products Act
ATG: antibody towards golimumab
CRF: case report form
CRP: C-reactive protein
DRKS: Deutsches Register Klinischer Studien, German Clinical Trials Register
GCP: good clinical practice
GLM: golimumab
IBD: inflammatory bowel disease
ICH: International Conference on Harmonization of Technical Requirements for Registration of Pharmaceuticals for Human Use
OR: odds ratio
TNFα: tumor necrosis factor alpha
UC: ulcerative colitis
WBC: white blood cells
